# Radiomic identification of anemia features in monochromatic conjunctiva photographs in school-age children

**DOI:** 10.1117/1.BIOS.2.2.022303

**Published:** 2025-04-15

**Authors:** Shaun G. Hong, Sang Mok Park, Semin Kwon, Haripriya Sakthivel, Jung Woo Leem, Steven R. Steinhubl, Pascal Ngiruwonsanga, Jean-Louis N. Mangara, Célestin Twizere, Young L. Kim

**Affiliations:** a Purdue University, Weldon School of Biomedical Engineering, West Lafayette, Indiana, United States; b Purdue University, Regenstrief Center for Healthcare Engineering, West Lafayette, Indiana, United States; c Rwanda Biomedical Center, Malaria, Neglected Tropical Diseases and Other Parasitic Diseases Division, Kigali, Rwanda; d University of Rwanda, Center of Excellence in Biomedical Engineering and eHealth, Kigali, Rwanda; e Purdue University, Purdue Institute for Cancer Research, West Lafayette, Indiana, United States; f Purdue University, Purdue Quantum Science and Engineering Institute, West Lafayette, Indiana, United States

**Keywords:** anemia, radiomics, smartphone, conjunctiva, mobile health, machine learning

## Abstract

**Significance:**

Anemia remains a substantial global health challenge. Delayed detection often leads to various health complications. In school-age children, anemia can impair both cognitive and physical development. Timely detection is particularly critical for this vulnerable population as effective interventions are available even in resource-limited settings.

**Aim:**

Most existing methods for assessing conjunctiva paleness or redness in anemia detection rely on colorimetric analyses or spectral imaging, which require sophisticated color processing methods or specialized equipment. We introduce an alternative that takes advantage of purely spatial and textural characteristics of the conjunctiva microvasculature for anemia detection.

**Approach:**

Radiomics, an emerging machine learning approach for conventional medical imaging, is applied to conjunctiva photos to analyze morphological alterations in the microvasculature beyond direct visualization. Radiomic analyses are conducted on 12,441 palpebral and 12,375 bulbar conjunctiva photos, captured using three different smartphone models from 565 children aged 5 to 15 years.

**Results:**

Spatial and textural features extracted from the palpebral and bulbar conjunctivae are significantly associated with anemia status in school-age children, demonstrating their potential as biomarkers of anemia.

**Conclusions:**

Instead of relying on color-based or spectral analyses of pallor in the conjunctiva, the proposed framework lays the groundwork for simplifying the hardware and algorithmic requirements of point-of-care, noninvasive anemia screening in sub-Saharan Africa and other resource-limited settings.

## 1 Introduction

Anemia, defined by reduced hemoglobin (Hgb) levels in the blood, is a major global health challenge affecting over 1.92 billion people worldwide with a disproportionate burden in low- and middle-income countries.^1,2^ Effective interventions for anemia include dietary modifications, supplementation (e.g., iron, folic acid, and vitamin B12), and blood transfusions for severe cases, even in resource-limited settings.^3–6^ Late or missed detection of anemia remains a primary challenge for effective screening and management in resource-limited settings. In particular, anemia in school-age children (aged 5 to 15 years) poses substantial health risks, including impaired cognitive and physical development, weakened immune function, and increased morbidity and mortality.^7–11^ Timely detection of anemia is particularly vital for this at-risk population.

The gold standard for diagnosing anemia involves clinical laboratory tests that measure Hgb levels or hematocrit in the blood from venous blood samples analyzed with a hematology analyzer.^12,13^ However, access to diagnostic testing in resource-constrained settings is often hindered by limited specialized equipment and clinical laboratory capacity. In response, point-of-care (POC) technologies for assessing blood Hgb levels or anemia have received considerable attention.^7,14–17^ Commercially available POC blood Hgb analyzers (e.g., Abbott i-STAT, HemoCue, and VERI-Q) rely on capillary blood sampling (finger-prick testing). However, these devices rely on environmentally sensitive cartridges with limited shelf lives.^18,19^ Commercially available noninvasive POC technologies (e.g., Masimo and OrSense) are available but require costly specialized equipment typically limited to advanced hospital settings.

Various mobile health (mHealth) technologies using optical imaging, spectroscopy, and smartphone cameras have been developed for noninvasive assessments of blood Hgb levels or anemia by sensing easily accessible peripheral tissue sites. Notable examples include smartphone-based sensing of the fingertip,^20,21^ smartphone photography of the fingernail,^22–24^ standalone fingertip devices,^25,26^ digital camera photography of the lip mucosa,^27^ and smartphone photography of the conjunctiva.^28–41^ Indeed, such noninvasive mHealth approaches have demonstrated the potential to enhance accessibility and reduce costs compared to traditional POC technologies. However, scalability and sustainability across diverse populations and clinical conditions remain key challenges requiring further research and development.

Inspired by clinical examinations for pallor in peripheral tissue, the conjunctiva is one of the common sites for assessing anemia. This easily accessible, noncontact imaging site reveals the underlying microvasculature, which is also unaffected by skin pigmentation (e.g., melanocytes).^28–41^ Nearly all existing methods of analyzing paleness or redness in the conjunctiva for anemia detection are based on red-green-blue (RGB) color analyses or spectral (hyperspectral or multispectral) imaging (Table S1 in the Supplementary Material).^28–41^ Spectral imaging systems, however, are bulky, expensive, and require specialized optical components. In addition, colorbased analyses are sensitive to environmental light conditions, smartphone models, and even file formats. Accurately and precisely extracting the true colors of a sample (i.e., achieving color accuracy) is a complex task that extends beyond white balancing (i.e., color constancy).^42,43^

We propose using the spatial and morphological characteristics of the conjunctiva as an alternative to anemia detection, eliminating the reliance on color or spectral analyses. Several studies have highlighted a relationship between anemia and microvascular changes in the conjunctiva. Although sickle cell anemia is manifested by microvascular alterations and abnormalities in the conjunctiva,^44,45^ other research has suggested a broader association between anemia and changes in microvascular morphology within the conjunctiva in general populations.^38,46–48^ Our approach aims to prioritize grayscale spatial analyses for anemia detection, motivated by the potential to bypass the need for bulky spectral imaging systems or complex color and hyperspectral signal processing.

However, the spatial resolution of conjunctiva images captured by an unmodified smartphone camera may be insufficient for directly quantifying the microvasculature in the conjunctiva. Radiomics, an emerging machine learning approach in biomedical imaging, has been extensively utilized in conventional optical and medical imaging modalities (e.g., optical coherence tomography, fundus photography, computed tomography, magnetic resonance imaging, and ultrasound).^49–52^ Radiomics facilitates the extraction of detailed spatial information on texture, shape, and intensity through a predefined set of mathematical characterizations beyond direct visualization.^49–53^ However, radiomics has not yet been widely applied in clinical settings or to smartphone photography. In this study, we hypothesize that radiomic features extracted from grayscale photos of the conjunctiva are associated with anemia status. We further investigate whether specific spatial and textural attributes from the palpebral and bulbar conjunctivae are comparable and can serve as potential biomarkers for anemia.

## 2 Methods

### 2.1 Study Design, Population, and Clinical Data Collection

This single-center, cross-sectional, observational study leveraged Rwanda’s healthcare system to collect data from 565 children aged 5 to 15 years at Gakoma District Hospital in Gisagara District, Rwanda. Gakoma District Hospital, located in Rwanda’s Southern Province, was chosen as the study site due to its partnerships with local schools. Children from nearby primary schools (ages 5 to 12) and junior secondary schools (ages 13 to 15) were invited to visit Gakoma District Hospital and participate in the study. Our study was part of an initiative to train school teachers as community health workers, recognizing their role as a vital resource to address gaps in healthcare services for school-age children as identified by the Rwanda Biomedical Center (implementation branch of the Ministry of Health).

The collected clinical data included sex, age, blood Hgb levels, and conjunctiva photos. Study nurses collected all clinical data within ~30 min per participant. As the gold standard for anemia diagnosis, blood Hgb levels were obtained from venous blood samples using a certified hematology analyzer (Sysmex XS-500i). The World Health Organization (WHO) definition of anemia was applied,^54,55^ using the following blood Hgb cutoffs: children aged 5 to 11 years (<11.5 gdL^−1^), children aged 11 to 14 years (< 12.0 gdL^−1^), nonpregnant women aged 15 years (<12.0 gdL^−1^), and men aged 15 years (<13.0 gdL^−1^). This clinical study was approved by the Rwanda National Ethics Committee (No. 93/RNEC/2023) and the Purdue University Institutional Review Board (No. IRB-2022–1644). Informed consent was obtained from a parent or guardian prior to participant enrollment.

### 2.2 Conjunctiva Photography Using Unmodified Smartphones

Multiple smartphone photos of the conjunctiva were captured immediately before or after venous blood draws for blood Hgb tests. Photos were acquired from both eyes and labeled as left and right using three different Android smartphone models (Samsung Galaxy S22, Samsung Galaxy A52, and Google Pixel 6). In particular, capturing multiple photos with different smartphone models enabled us to assess the generalizability across various devices. The photo acquisition protocol accounted for diverse conditions in a real-world setting; the light conditions were ambient room light or sunlight without the use of a smartphone flashlight. Our study personnel captured photos, while the volunteer participant pulled down the eyelid to expose the conjunctiva, including the palpebral and bulbar conjunctivae. The photos were time-stamped, saved in JPEG format, and assigned a unique participant identifier. Although radiomic analyses did not rely on direct quantification of spatial features, we estimated the spatial resolution of the cameras in three different smartphone models: Samsung Galaxy S22, Samsung Galaxy A52, and Google Pixel 6. We used the edge method,^56,57^ considering an average distance of 100 to 150 mm between the camera and the participant’s conjunctiva. The spatial resolution was determined by the full width at half maximum of the line spread function derived from the edge spread function, resulting in 172 *μ*m for Samsung Galaxy S22, 110 *μ*m for Samsung Galaxy A52, and 137 *μ*m for Google Pixel 6, respectively.

### 2.3 Characteristics of the Study Participants and Clinical Data

Table 1 summarizes the overall characteristics of the study participants. The radiomic analyses for anemia prediction utilized 12,441 photos of the palpebral conjunctiva and 12,375 photos of the bulbar conjunctiva from 565 children aged 5 to 15 years who visited Gakoma District Hospital in Gisagara District, Rwanda. The photo data were divided into training (70% of participants) and testing (30%) datasets based on participant identifiers as anemia diagnostics were conducted at the individual level. The photos from the same participants were assigned exclusively to either the training or testing datasets to prevent overlap. Specifically, a stratified random split was employed to maintain a proportional representation of anemic and nonanemic cases within each subset.^58,59^ This approach preserves the distribution of the outcome variable in both groups, reducing class imbalances. The training and testing datasets for both the palpebral and bulbar conjunctivae exhibited statistically similar trends, including age, sex, blood Hgb levels, and anemia status. It should be noted that we utilized conjunctiva segmentation models independently trained with transfer learning and an anemia classification model with the hyperparameters optimized on a small subset of the training dataset. The testing dataset was exclusively used to evaluate the performance of radiomic analyses and anemia prediction, ensuring no data leakage.

### 2.4 Conjunctiva Segmentation and Annotation

We used an automated segmentation model to automatically delineate the palpebral and bulbar conjunctivae separately. Specifically, we employed Mask R-CNN for instance segmentation, implemented with Detectron2, an open-source library developed by Facebook AI Research.^60^ We utilized a pretrained model from the Detectron2 library, trained on the “Common Objects in Context” (COCO) dataset, which contains over 330,000 images and 2.5 million object instances.^61^ For region-of-interest annotation, we used LabelMe, an image labeling tool that provides a graphical, polygonal annotation function for precisely defining object edges. The annotated images for training Mask R-CNN were cross-validated to ensure consensus on the various shapes of the palpebral and bulbar conjunctivae. The Mask R-CNN segmentation model was trained independently as transfer learning proved sufficient for this task, by utilizing approximately 100 photos that were not part of the main datasets. When compared with manually segmented results, the automated segmentation model achieved a mean Dice similarity coefficient of 0.92 ± 0.03 (standard deviation) for the palpebral conjunctiva and 0.91 ± 0.04 for the bulbar conjunctiva. This level of performance ensured reliable region delineation for radiomic analyses.

### 2.5 Grayscale Image Conversion from RGB Images

We converted the images to grayscale to test the hypothesis without the influence of color variations, which are often affected by smartphone models and light conditions. Specifically, we employed a method that calculates a single intensity value for each pixel by combining the RGB color channel values, resulting in a balanced grayscale representation. After conversion, we standardized the images by adjusting the brightness values to a fixed range.

### 2.6 Radiomic Feature Extractions

We extracted radiomic features relevant to anemia for the palpebral and bulbar conjunctivae, using predefined configuration parameters. We followed the guidelines of radiomics standardization.^62^ The analyses focused on three types of radiomic features: (1) first-order statistics describing intensity distributions, (2) textural features capturing spatial relationships, and (3) wavelet features derived from wavelet transformation. Shape-related features were excluded as the shapes of the palpebral and bulbar conjunctivae are easily changed by how individuals open their eyes and pull down their eyelids. In general, radiomics generates a large number of spatial and textural features, which is a key strength that qualifies it as an “omics” method. In this study, we initially extracted 1078 radiomic features from the conjunctiva region before applying feature selection. To ensure comparability across images, all extracted radiomic features were standardized using *Z*-score normalization. It is important to note that radiomic feature selection was performed exclusively on the training dataset.

### 2.7 Radiomic Feature Selections

We selected radiomic features relevant to anemia from the palpebral and bulbar conjunctivae separately, following the handling of missing values and the application of min-max scaling. Missing values were imputed using the *k*-nearest neighbors algorithm. The features were scaled to a range between 0 and 1 to ensure equal contributions across all features. For feature ranking, we used random forest classifiers trained iteratively using only the training dataset. In each iteration, feature importance was calculated, and the top features were identified based on their contributions to the classification task. Importantly, we limited the number of radiomic features used to mitigate the risk of overfitting when building the anemia classification model. We selected a small subset of radiomic features that demonstrated high consistency across multiple iterations of feature ranking using the random forest algorithm. Specifically, 20 radiomic features consistently ranked among the top across different random seeds. Only features that consistently ranked high across different random seeds were retained to ensure the selection of the most relevant ones. A conservative selection of 20 features was finalized in each case of the palpebral and bulbar conjunctivae. This approach also ensured a balance between model interpretability and classification performance while minimizing overfitting.

### 2.8 Classification Model Using Neural Networks

We constructed neural network classification models to predict anemia using the selected radiomic features from the palpebral and bulbar conjunctivae separately. Each fully connected neural network consisted of an input layer with 20 nodes, two hidden layers with 50 nodes each, and an output layer for binary classification of anemia status. The rectified linear unit activation function was applied in the hidden layers. The training was conducted using the adaptive moment estimation (ADAM) optimizer. Hyperparameter tuning involved a grid search to identify the optimal learning rate, batch size, and number of epochs. Specifically, 20% of the training dataset was allocated for a randomized search to optimize hyperparameters. To address the class imbalance, where nonanemic participants outnumbered anemic ones, the synthetic minority oversampling technique (SMOTE) was applied to create a balanced dataset, improving the model’s generalization and reducing bias toward the majority class.^63^

### 2.9 Statistical Analyses

We summarized normally distributed continuous variables using means and standard deviations, whereas nonnormally distributed variables were summarized using medians and interquartile ranges. For categorical variables, we applied the chi-square test or Fisher’s exact test as appropriate. To evaluate the performance of the classification model, we generated receiver operating characteristic (ROC) curves and calculated the area under the curve (AUC). When comparing two classification models, we used DeLong’s test, a nonparametric statistical method that accounts for data correlation when analyzing differences between two ROC curves. For comparisons involving more than two classification models, we used the Kruskal–Wallis test to analyze AUC values across different models. In all cases, a *p*-value threshold of <0.05 was considered statistically significant. All statistical analyses were performed using Python (SciPy and Statsmodels) or R (pROC package for ROC comparisons).

## 3 Results

### 3.1 Flowchart of Mobile Health (mHealth) Radiomic Analyses

**Fig. 1:**
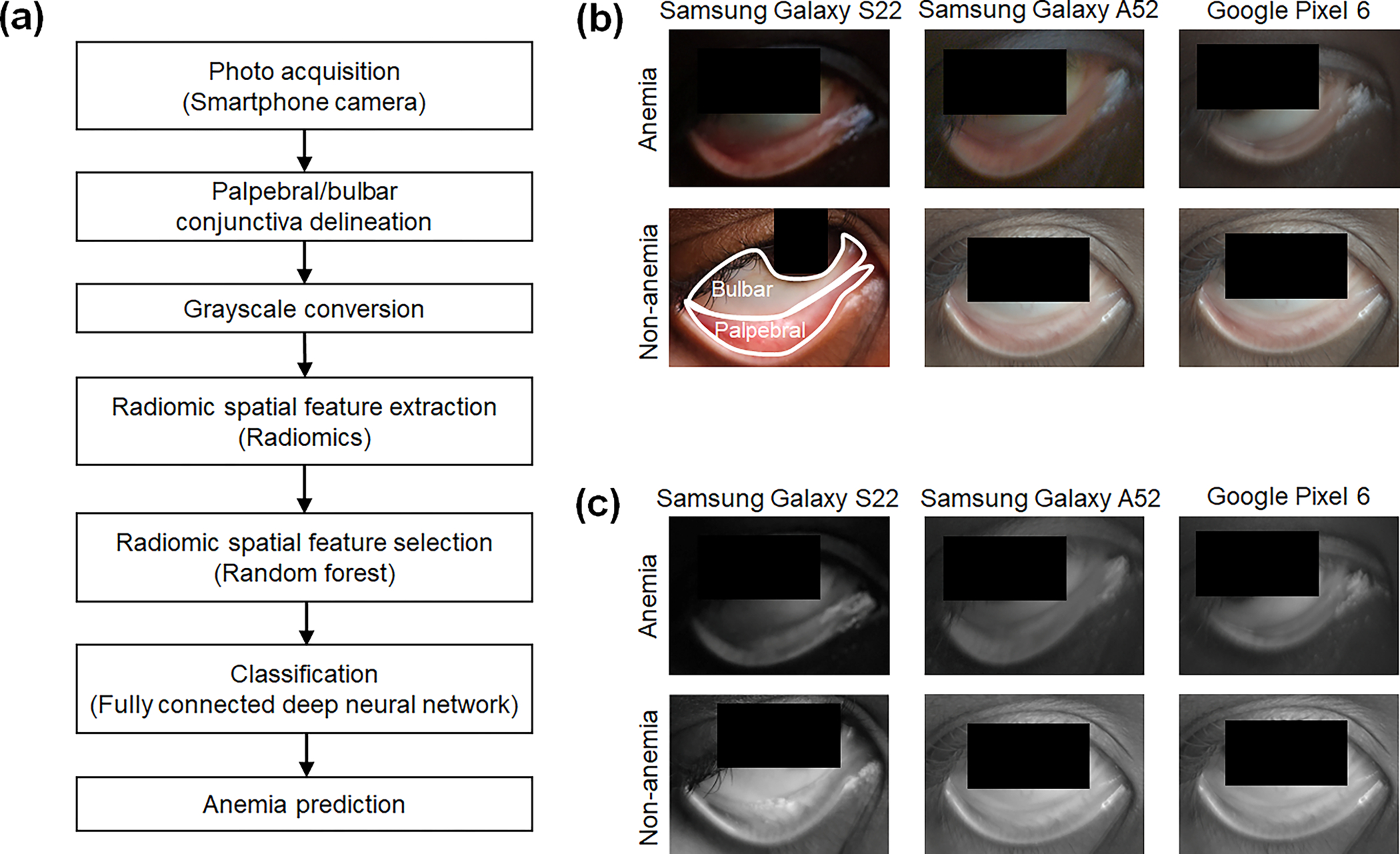
mHealth radiomic analyses of grayscale conjunctiva photographs for anemia prediction. (a) Flowchart of the radiomic analyses for anemia prediction, conducted separately for the palpebral and bulbar conjunctivae. Each analysis focuses on extracting predefined mathematical characterizations of spatial and textural patterns associated with anemia. (b) Representative photos of the palpebral and bulbar conjunctivae for anemia and nonanemia, captured using three different Android smartphone models: Samsung Galaxy S22, Samsung Galaxy A52, and Google Pixel 6. The palpebral and bulbar conjunctivae are automatically delineated with a deep learning–based segmentation model using Mask R-CNN. (c) Corresponding monochromatic grayscale images converted from the original RGB photos are used for the radiomic analyses. Monochromatic grayscale smartphone photos of accessible peripheral tissue (conjunctiva) eliminate the need for color correction or white balancing, which can vary across smartphone models and light conditions.

Figure 1(a) illustrates the overall workflow of the proposed radiomic analyses. The input data consist of multiple photos captured from both eyes of each participant using three different smartphone models: Samsung Galaxy S22, Samsung Galaxy A52, and Google Pixel 6 in Sec. 2. A deep learning segmentation model based on Mask R-CNN is used to automatically delineate two distinct regions of the conjunctiva: the palpebral conjunctiva and the bulbar conjunctiva. To reduce color variability caused by different light conditions and model-specific spectral sensitivity functions,^64,65^ the RGB photos are converted to grayscale. Radiomic feature extraction focuses on identifying spatial and textural patterns using predefined mathematical representations in accordance with the radiomics standardization guidelines.^62^ Radiomic feature selection identifies important features associated with anemia by ranking them using random forest classifiers. Classification models for predicting anemia from the palpebral and bulbar conjunctivae are separately developed using fully connected neural networks with the selected radiomic features as inputs.

### 3.2 Grayscale Conjunctiva Smartphone Photos

Spatial and textural radiomics provides alternatives to color-based or spectral analyses that assess the paleness of peripheral tissue sites including the conjunctiva. In our study, the color-independent conjunctiva photos form the basis for testing the hypothesis that spatial features and microvascular changes in the conjunctiva are indicative of anemia. Figure 1(b) illustrates representative photos of anemic and nonanemic individuals, captured using three different smartphone models. The inclusion of photos from multiple devices underscores the adaptability and robustness of the reported analyses across varying hardware and light conditions. The clearly delineated palpebral and bulbar regions further demonstrate the performance of Mask R-CNN segmentation. Subsequently, the grayscale images are utilized to capture texture and spatial characteristics associated with anemia status [Fig. 1(c)]. This independence from color information eliminates the need for color correction or white balancing, which are challenging to implement as universal methods applicable across diverse conditions.

### 3.3 Radiomic Features and Characteristics Relevant to Anemia

**Fig. 2:**
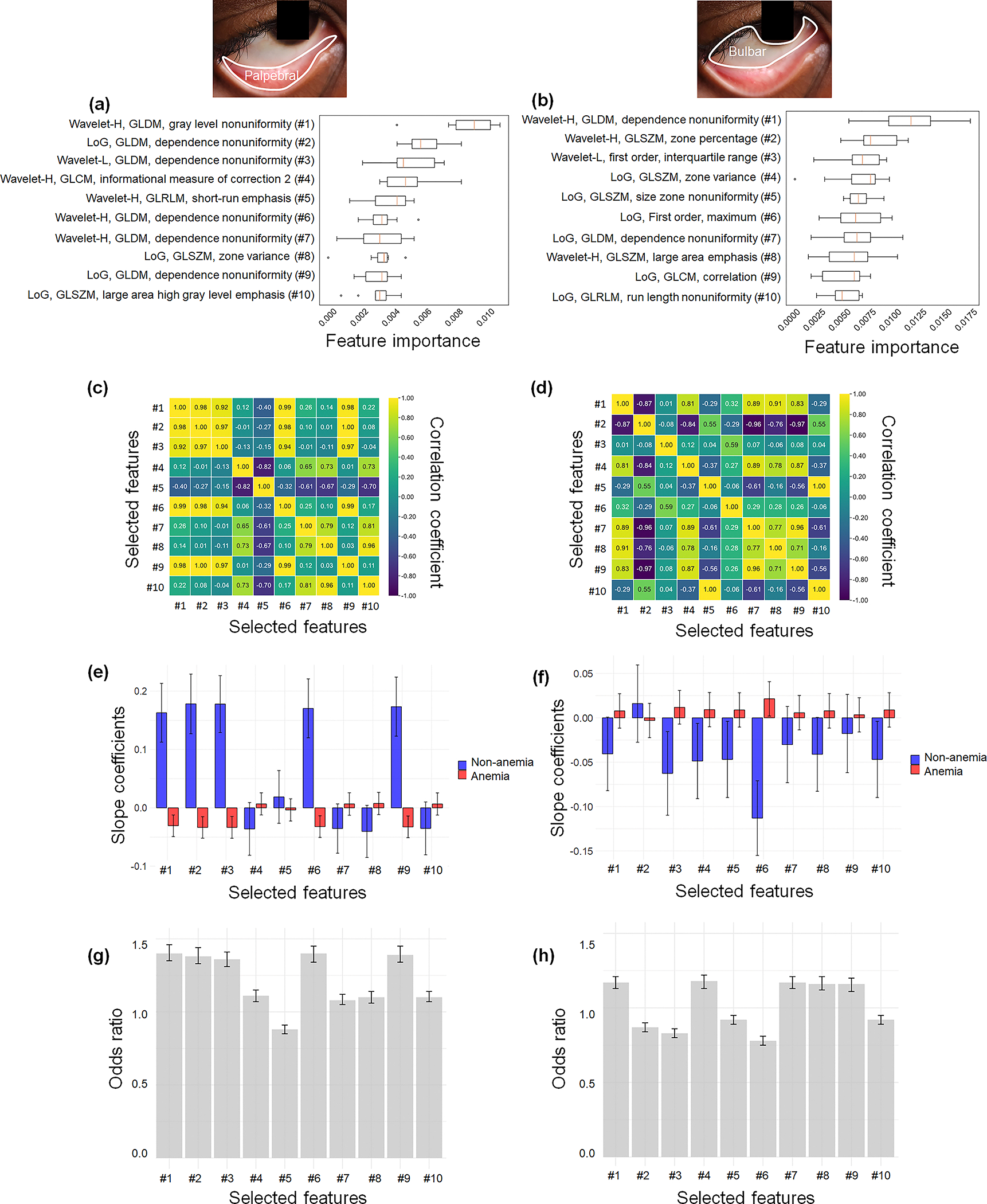
Identification of radiomic features relevant to anemia and correlation analyses in the palpebral and bulbar conjunctivae for anemia prediction. (a) and (b) Feature importance of the top 10 radiomic features, including first-order statistics, textural features, and wavelet features for the palpebral (a) and bulbar (b) conjunctivae. The insets show the corresponding delineated regions (palpebral versus bulbar conjunctivae). The bar graph displays the mean decrease in impurity (also known as Gini importance) for each feature. (c) and (d) Pairwise correlation coefficients among the selected radiomic features for the palpebral (c) and bulbar (d) conjunctivae. The heatmap reveals the absence of perfect unity among the coefficients, underscoring the uniqueness of each selected feature. (e) and (f) Differences in slope coefficients from multivariate linear regression for distinguishing between anemia and nonanemia for the palpebral (e) and bulbar (f) conjunctivae. The regression is performed using the selected features to predict blood Hgb levels as a numerical output. The slope coefficients are plotted separately for anemia and nonanemia. The error bars represent the 95% confidence intervals. (g) and (h) Odds ratios from multivariate logistic regression for distinguishing between anemia and nonanemia for the palpebral (g) and bulbar (h) conjunctivae. The regression is performed using the selected features to predict the anemia status. The error bars represent the 95% confidence intervals.

Figures 2(a) and 2(b) show representative important radiomic features in the palpebral and bulbar conjunctivae, respectively, associated with anemia status. Each ranking is determined using the mean decrease in impurity (also known as Gini importance) scores. Although the selected radiomic features differ between the palpebral and bulbar conjunctivae, the features of high-pass wavelet (Wavelet-H) and gray level dependence matrix (GLDM) are prominent in both conjunctivae (Tables S2 and S3 in the Supplementary Material). Other common features include Laplacian of Gaussian (LoG), low-pass wavelet (Wavelet-L), GLDM, gray level co-occurrence matrix (GLCM), gray level run length matrix (GLRLM), and gray level size zone matrix (GLSZM) (Tables S2 and S3 in the Supplementary Material). In Figs. 2(c) and 2(d), the pairwise correlation analyses of the selected radiomic features show that the correlation coefficients among these features are neither perfectly positive nor perfectly negative, indicating their unique contributions to anemia detection.

Multivariate linear regression [Figs. 2(e) and 2(f) and Table S4 in the Supplementary Material] demonstrates that the top 10 features are distinct between anemic and nonanemic groups, when the selected radiomic features are used as input variables and blood Hgb levels as the numerical output variable. Figures 2(e) and 2(f) highlight the slope differences in the top 10 radiomic features between the two groups. Almost all of these top 10 features are statistically significantly associated with blood Hgb levels in both the palpebral and bulbar conjunctivae (Table S4 in the Supplementary Material). Multivariate logistic regression [Figs. 2(g) and 2(h) and Table 2] directly captures the associations between the selected radiomic features with anemia status. More importantly, none of the odds ratios for the top 10 selected radiomic features are close to unity, with all *p*-values of <0.05. These results support the idea that the selected radiomic features uniquely contribute as input variables for anemia prediction. In addition, the selected radiomic features in the palpebral and bulbar conjunctivae highlight the distinct texture and spatial variability of the conjunctiva in relation to anemia.

### 3.4 Performance of Predicting Anemia Across Subgroups

**Fig. 3:**
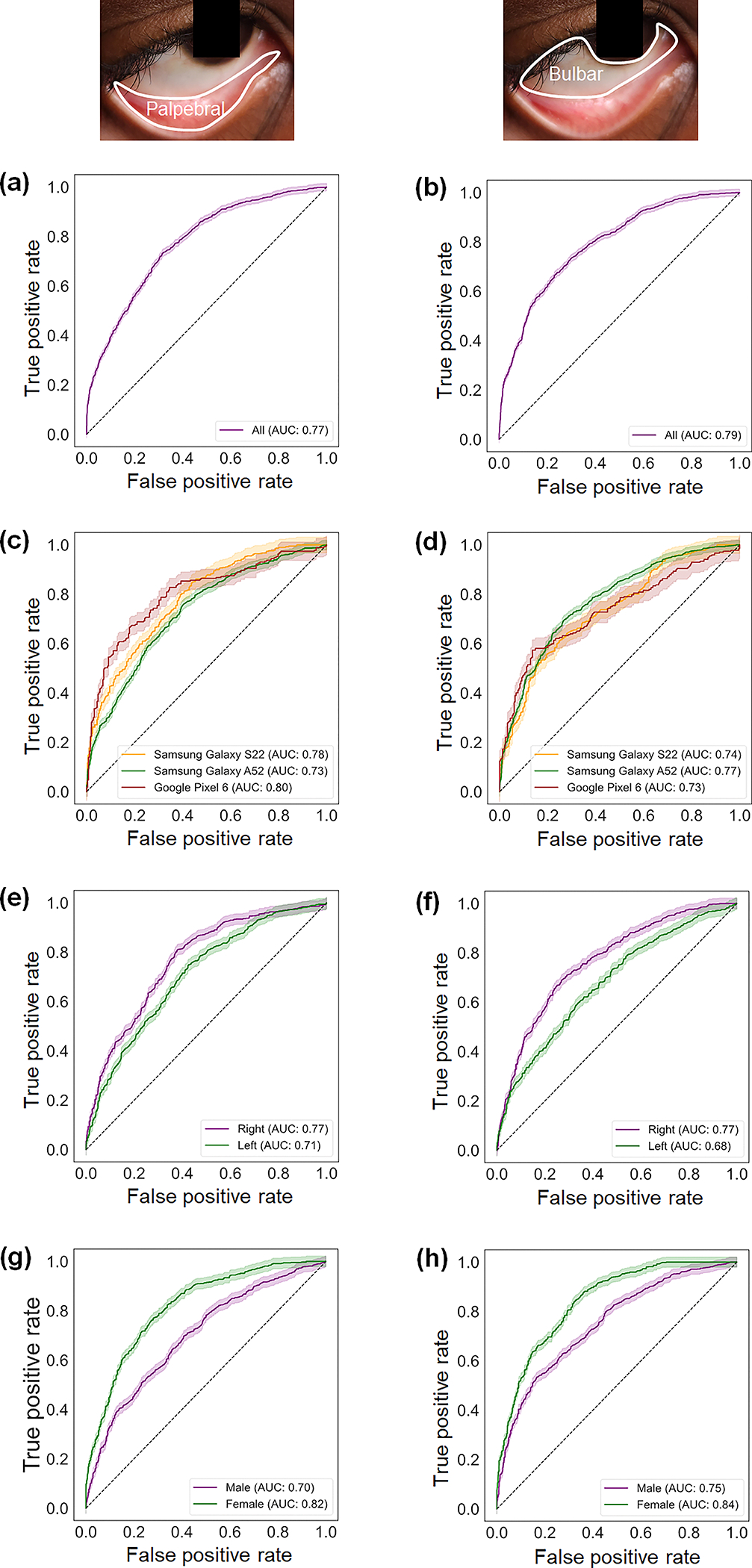
Performance of selected radiomic features in the palpebral and bulbar conjunctivae for anemia prediction. (a) and (b) ROC curves of the anemia prediction models plotted for positive rates for the palpebral (a) and bulbar (b) conjunctivae. The 95% confidence intervals are calculated based on the Wald intervals. The insets show the corresponding delineated regions (palpebral versus bulbar conjunctivae). AUC represents the area under the ROC curve. (c) and (d) ROC curves of the anemia prediction models plotted for positive rates across three different smartphone models (Samsung Galaxy S22, Samsung Galaxy A52, and Google Pixel 6) for the palpebral (c) and bulbar (d) conjunctivae. (e) and (f) ROC curves of the anemia prediction models plotted for positive rates in the right and left eyes for the palpebral (e) and bulbar (f) conjunctivae. (g) and (h) ROC curves of the anemia prediction models plotted for positive rates across different sexes (male and female) for the palpebral (g) and bulbar (h) conjunctivae.

Using the selected radiomic features exclusively from the testing dataset of the palpebral and bulbar conjunctivae, Figs. 3(a) and 3(b) present ROC curve analyses to illustrate the performance of the neural network-based anemia classification models. For the palpebral conjunctiva, the model’s ability to distinguish between anemic and nonanemic groups achieves an AUC of 0.77 with a 95% confidence interval (CI) ranging from 0.76 to 0.79, calculated exclusively using the testing dataset [Fig. 3(a)]. For the bulbar conjunctiva, the model’s ability to distinguish between anemic and nonanemic groups achieves an AUC of 0.79 with a 95% CI ranging from 0.78 to 0.81, calculated exclusively using the testing dataset [Fig. 3(b)]. When comparing the models between the palpebral and bulbar conjunctivae, the ROC curves are not statistically different as supported by the DeLong test with a *p*-value of 0.40.

Despite differences in onboard camera specifications among the three smartphone models, the classification models are not dependent on the specific smartphone used. The ROC curves for the palpebral conjunctiva (*p*-value of Kruskal–Wallis test = 0.368) and bulbar conjunctiva (*p*-value of Kruskal–Wallis test = 0.368) are not statistically different among the smartphone models [Figs. 3(c) and 3(d)]. In addition, the spatial and textural variability captured by the radiomic features can be applied universally to either eye. The ROC curves between the left and right eyes are also not statistically different for the palpebral conjunctiva (*p*-value of DeLong test = 0.618) and the bulbar conjunctiva (*p*-value of DeLong test = 0.647) [Figs. 3(e) and 3(f)]. On the other hand, sex-based analyses reveal higher AUCs for females (AUC = 0.82 for the palpebral conjunctiva and AUC = 0.84 for the bulbar conjunctiva) compared with males (AUC = 0.70 for the palpebral conjunctiva and AUC = 0.75 for the bulbar conjunctiva) [Figs. 3(g) and 3(h)]. These differences are statistically significant for both the palpebral conjunctiva (*p*-value of DeLong test <0.001) and the bulbar conjunctiva (*p*-value of DeLong test = 0.004).

## 4 Discussion

The reported radiomic machine learning analyses of spatial and textural attributes in conjunctiva photos provide two key advantages over other noninvasive anemia detection methods, which rely on color-based or spectral quantification of pallor. First, by leveraging the radiomic analyses of conjunctiva photos, the spatial and texture characteristics reflecting microvascular alterations in the conjunctiva can be used to differentiate between anemic and nonanemic cases. Second, the combination of radiomics and the fully connected neural network for anemia classification provides a certain level of interpretability and explainability. The fully connected neural network for the anemia classification models consists of layers in which each node is connected to every node in the next layer, making it more suitable for structured/tabular data (radiomic features). The fully connected neural network is analogous to statistical regression because it can incorporate structured/tabular data. This transparency facilitates multivariate statistical analyses by integrating radiomic features with nonradiomic clinical data.^49–53^

This study focuses on school-age children aged 5 to 15 years, a population for whom early and timely detection of anemia is especially critical. Untreated anemia can impair cognitive and physical development.^7–11^ In low- and middle-income countries, the prevalence of anemia is disproportionately high due to factors including nutrient deficiencies, infectious diseases, and hemoglobinopathies.^66^ Anemia in children is associated with health risks, including weakened immune function and increased morbidity and mortality.^10^ Delayed transfusion in severely anemic children increases mortality, emphasizing the need for prompt detection.^10^ Early anemia detection enables timely intervention, improves health outcomes, and supports children’s overall growth and development.

The prediction performance of anemia classification models is statistically higher for females than for males in both the palpebral and bulbar conjunctivae, potentially suggesting sex-specific variations in the characteristics associated with anemia status. The observed higher AUC in females when using conjunctival photos for anemia classification may be attributed to: higher capillary density, making microvascular patterns more distinct; greater endothelial reactivity, enhancing microvascular contrast; and smaller vessel diameter, potentially making subtle variations more detectable.^67,68^ Regarding the left and right eyes, the ROC curves for the palpebral and bulbar conjunctivae do not show a statistically significant difference between the left and right eyes, indicating that either eye can be used. However, more detailed and extensive studies focusing on conjunctiva microvasculature using advanced imaging methods are warranted.

Capturing conjunctiva photos using an unmodified smartphone and analyzing their spatial features offers a practical and accessible solution for fieldwork and applications in resource-limited settings. Radiomic analysis effectively captures both fine and coarse image details, presenting an advantage over traditional color-based or spectral imaging methods, which often rely on complex color correction or specialized equipment. This quantitative, color-independent framework is expected to enable the development of a simple and computationally efficient algorithm for anemia screening, agnostic to diverse light conditions and smartphone models.

Given the potential applications in resource-limited settings, cost and affordability are key considerations in the selection criteria for smartphones. In Africa, Android smartphones are more prevalent than iOS devices (Apple products).^69^ In our study, the Samsung Galaxy S22, Google Pixel 6, and Samsung Galaxy A52 represent three distinct price tiers, ranging from high-end to mid-range and budget-friendly options. Differences in onboard camera specifications and features across these smartphone models lead to variations in color tones, contrast, and signal-to-noise ratios. Thus, ensuring generalizability across a diverse range of smartphone models is crucial. The reported radiomic anemia detection remains relatively agnostic to these three smartphone models—a critical factor in mHealth research for real-world implementation.

One limitation of the current approach is that it predicts a binary outcome for anemia. Diagnostic applications frequently involve outcomes with ordered categorical levels to guide appropriate clinical intervention strategies. For example, the WHO guidelines classify anemia into three categories: mild, moderate, and severe anemia, based on factors such as age, sex, and pregnancy status.^54,55^ On the other hand, machine learning and statistical regression methods often struggle to effectively discriminate between ordered polytomous outcomes, particularly for model assessment and evaluation of ordinal outcomes. Our ongoing efforts to enhance performance include combining color-based analyses (pallor) with radiomic analyses (spatial and textural information). We are developing advanced color correction and white balancing methods. In addition, expanding the dataset to include a broader range of blood Hgb levels will improve the prediction performance.

## 5 Conclusion

By leveraging spatial and textural attributes in smartphone photos of the conjunctiva instead of color-based or spectral analyses, this study establishes a foundation for an alternative approach to anemia detection, emphasizing its clinical relevance for straightforward anemia screening. Radiomic features extracted from the palpebral and bulbar conjunctivae show strong correlations with anemia status in school-age children in sub-Saharan Africa. The palpebral and bulbar conjunctivae are both accessible and comparable sites for anemia prediction. This framework, which relies solely on smartphone cameras without requiring additional hardware or complex algorithms, addresses the hardware and computational challenges associated with conventional anemia diagnostics. Once a fully functional mobile app is developed, this mHealth solution could prove particularly valuable for population-level screening or prescreening in resource-limited settings, where limited access to clinical laboratories and trained medical personnel often delays timely anemia detection.

## Supplementary Material

Supplementary Material

## Figures and Tables

**Table 1:** Overall characteristics of the study population, training dataset, and testing dataset for the palpebral and bulbar conjunctivae.

	Total	Training	Testing	*p*-Value

**Palpebral conjunctiva**				
Number of photos	12,441	8708	3733	—
Number of participants	565	395	170	—
Age [years] (mean ± standard deviation)	10.48 ± 3.06	10.36 ± 3.07	10.76 ± 3.02	0.152
Sex [males] (%)	300 (53.10%)	207 (52.41%)	93 (54.71%)	0.681
Blood hemoglobin [g dL^−1^] (mean ± standard deviation)	10.28 ± 1.66	10.22 ± 1.67	10.41 ± 1.63	0.194
Anemia (%)	56.21	55.89	56.95	0.275
**Bulbar conjunctiva**				
Number of photos	12,375	8829	3546	—
Number of participants	565	395	170	—
Age [years] (mean ± standard deviation)	10.48 ± 3.06	10.49 ± 3.08	10.46 ± 3.02	0.915
Sex [males] (%)	300 (53.10%)	201 (50.89%)	99 (58.24%)	0.130
Blood hemoglobin [g dL^−1^] (mean ± standard deviation)	10.28 ± 1.66	10.28 ± 1.67	10.27 ± 1.65	0.941
Anemia (%)	56.20	56.62	55.24	0.157

*p*-Values are calculated using *t*-tests between the training and testing datasets.

**Table 2:** Multivariate logistic regression of associations between anemia and selected radiomic features.

	Anemia	

	Odds ratio (*p*-value)	95% confidence interval

**Palpebral conjunctiva**		

Wavelet-H, GLDM, gray level nonuniformity (#1)	1.40 (0.000)	1.35, 1.46
LoG, GLDM, dependence nonuniformity (#2)	1.38 (0.000)	1.33, 1.44
Wavelet-L, GLDM, dependence nonuniformity (#3)	1.36 (0.000)	1.31, 1.41
Wavelet-H, GLCM, informational measure of correction 2 (#4)	1.11 (0.000)	1.07, 1.15
Wavelet-H, GLRLM, short run emphasis (#5)	0.88 (0.000)	0.85, 0.91
Wavelet-H, GLDM, dependence nonuniformity (#6)	1.40 (0.000)	1.34, 1.45
Wavelet-H, GLDM, dependence nonuniformity normalized (#7)	1.08 (0.000)	1.05, 1.12
LoG, GLSZM, zone variance (#8)	1.10 (0.000)	1.06, 1.14
LoG, GLDM, dependence nonuniformity (#9)	1.39 (0.000)	1.34, 1.45
LoG, GLSZM, large area high gray level emphasis (#10)	1.10 (0.000)	1.07, 1.14

**Bulbar conjunctiva**		

Wavelet-H, GLDM, dependence nonuniformity normalized (#1)	1.17 (0.000)	1.13, 1.21
Wavelet-H, GLSZM, zone percentage (#2)	0.87 (0.000)	0.84, 0.9
Wavelet-L, first order, interquartile range (#3)	0.83 (0.000)	0.80, 0.86
LoG, GLSZM, zone variance (#4)	1.18 (0.000)	1.13, 1.22
LoG, GLSZM, size zone nonuniformity normalized (#5)	0.92 (0.000)	0.89, 0.95
LoG, first order, maximum (#6)	0.78 (0.000)	0.75, 0.81
LoG, GLDM, dependence nonuniformity normalized (#7)	1.17 (0.000)	1.13, 1.21
Wavelet-H, GLSZM, large area emphasis (#8)	1.16 (0.000)	1.12, 1.21
LoG, GLCM, correlation (#9)	1.16 (0.000)	1.11, 1.2
LoG, GLRLM, run length nonuniformity normalized (#10)	0.92 (0.000)	0.89, 0.95

Wavelet-H, high-pass wavelet; Wavelet-L, low-pass wavelet; LoG, Laplacian of Gaussian; GLDM, gray level dependence matrix; GLCM, gray level co-occurrence matrix; GLRLM, gray level run length matrix; GLSZM, gray level size zone matrix.

## Data Availability

The data that support the findings of this study are included in the main text and Supplementary Material. The computational models and algorithms used in this study rely on standard toolboxes and libraries that are publicly available in MATLAB and Python.

## Statement of Discovery

This study demonstrates that spatial and textural radiomic features in photos of the eye and eyelids (conjunctiva) are significantly associated with anemia status. A color-free monochromatic machine learning approach paves the way for developing a noninvasive, affordable, and scalable mobile health (mHealth) application capable of detecting anemia using off-the-shelf smartphones and straightforward computation.
