# Supplementary material for: Radiomic identification of anemia features in monochromatic conjunctiva photographs in school-age children
Source: Biophotonics Discov. Author manuscript; Available in PMC 2025 Jun 18. (PMC12176424; doi:10.1117/1.BIOS.2.2.022303)
Supplement: Supplementary file 1 [file NIHMS2085415-supplement-Supplementary_Material.pdf]

Supplementary Material

Radiomic identification of anemia features in monochromatic conjunctiva photographs in school-age children

Shaun G. Hong[1], Sang Mok Park[1], Semin Kwon[1], Haripriya Sakthivel[1], Jung Woo Leem[1], Steven R. Steinhubl[1,2], Pascal Ngiruwonsanga[3], Jean-Louis N. Mangara[3], Célestin Twizere[4], and Young L. Kim[1,2,5,6*]
*youngkim@purdue.edu

[1]Weldon School of Biomedical Engineering, Purdue University, West Lafayette, Indiana, USA
[2]Regenstrief Center for Healthcare Engineering, Purdue University, West Lafayette, Indiana, USA
[3]Malaria, Neglected Tropical Diseases and Other Parasitic Diseases Division, Rwanda Biomedical Center, Kigali, Rwanda
[4]Center of Excellence in Biomedical Engineering and eHealth, University of Rwanda, Kigali, Rwanda
[5]Purdue Institute for Cancer Research, Purdue University, West Lafayette, Indiana, USA
[6]Purdue Quantum Science and Engineering Institute, Purdue University, West Lafayette, Indiana, USA

Supplementary Table S1. Summary of conjunctiva photo-based technologies for assessing blood hemoglobin (Hgb) levels or anemia

Anatomical site	Primary outcome	Requirement of color or spectral information	Main algorithm	Separate testing or validation	Validation across different devices (smartphones)	Reference
Palpebral conjunctiva	Hgb level	Yes	Stochastic modeling of light propagation through multilayered human palpebral conjunctiva tissue using Monte Carlo simulations	Yes	No	Kim et al. (2014) [28]
Palpebral conjunctiva	Anemia	Yes	Erythema index with red and green color analyses	Yes	Yes	Collings et al. (2016) [70]
Palpebral conjunctiva	Hgb level	Yes	Partial least squares regression on spectral reconstruction from RGB	Yes	No	Kim et al. (2016) [29]
Palpebral conjunctiva	Anemia	Yes	Simple thresholding and a Mahalanobis distance classifier based on color features as well as support vector machines and neural networks	Yes	No	Chen et al. (2016) [71]
Palpebral conjunctiva	Hgb level	Yes	Kalman filtering with nonlinear penalty regression on RGB data	Yes	No	Chen et al. (2017) [72]
Palpebral conjunctiva	Anemia	Yes	Simple linear iterative clustering superpixel segmentation	Yes	Yes	Dimauro et al. (2019) [30]
Palpebral conjunctiva	Hgb level	Yes	Ridge regression using red and green color information	Yes	No	Kasiviswanathan et al. (2020) [73]
Palpebral conjunctiva	Hgb level	Yes	Hyperspectral reconstruction from RGB data and partial least squares regression	Yes	No	Park et al. (2020) [31]
Palpebral conjunctiva	Hgb level	Yes	Smartphone color spectroscopy on conjunctiva pallor	Yes	No	Ghosal, et al. (2021) [32]
Palpebral conjunctiva	Hgb level	Yes	Stepwise regression with conjunctiva color feature extraction	Yes	Yes	Suner et al. (2021) [33]
Palpebral conjunctiva	Anemia	Yes	Convolutional neural network, logistic regression, gaussian blur on conjunctiva color features	Yes	Yes	Appiahene et al. (2023) [74]
Palpebral conjunctiva	Hgb level	No	Convolutional neural network combining demographic and image data	Yes	No	Çuvadar et al. (2023) [47]
Palpebral conjunctiva	Anemia	Yes	Random undersampling and boosting machine learning for color feature extraction	Yes	Yes	Dimauro et al. (2023) [39]
Bulbar conjunctiva	Anemia	Yes	Polynomial support vector machine classifier for anemia detection based on scleral and vessel color features	Yes	No	Dimauro et al. (2023) [75]
Palpebral conjunctiva	Hgb level	No	Mask region-based convolutional neural network for feature extraction	Yes	No	Hu et al. (2023) [38]
Palpebral conjunctiva	Anemia	Yes	High hue ratio as red-green hue analyses	Yes	Yes	Zhao et al. (2024) [40]
Palpebral conjunctiva	Anemia	No	Convolutional neural network, segmentation, and classification without explicit color focus	Yes	Yes	Pallavi et al. (2024) [48]
Palpebral conjunctiva	Anemia	Yes	Ensemble model (k-nearest neighbors, random forest, decision tree).	Yes	No	Kasiviswanathan et al. (2024) [76]
Palpebral conjunctiva	Hgb level	Yes	Color intensity analyses and binary encoding	Yes	No	Chen et al. (2024) [41]

Supplementary Table S2. High-level categories of selected radiomic features in conjunctiva photos for anemia

High-level category	Description	Mathematical definition
High-pass wavelet (Wavelet-H)	Extracts high-frequency details (e.g., edges, sharp transitions, and noise) by decomposing the image into wavelet coefficients through the application of high-pass filters.	Wavelet-H decomposes the image into coefficients representing sharp transitions or edges by applying a wavelet transform that isolates high-frequency details.
Low-pass wavelet (Wavelet-L)	Preserves low-frequency details (e.g., smooth regions and overall intensity patterns) by decomposing the image into wavelet coefficients through the application of low-pass filters.	Wavelets-L decomposes the image into coefficients representing smooth regions and overall intensity patterns by applying a wavelet transform that filters out high-frequency components.
Laplacian of Gaussian (LoG)	Detects edges by highlighting regions of rapid intensity change, combining Gaussian smoothing to reduce noise with the Laplacian operator, often making it useful for identifying blob-like structures.	LoG is calculated by first applying a Gaussian filter to smooth the image, reducing noise, and then applying the Laplacian operator to detect edges by identifying areas of rapid intensity change. The Gaussian filter is governed by a parameter that defines the scale of smoothing.
Gray level dependence matrix (GLDM)	Captures spatial dependency by the number of neighboring pixels within a specified gray-level intensity difference, enabling texture analysis that is sensitive to subtle variations.	GLDM is constructed by counting the number of pixels with a specific gray level that have a defined number of neighboring pixels (dependencies) within a specified intensity difference threshold.
Gray level co-occurrence matrix (GLCM)	Quantifies texture by examining the spatial relationship between pairs of pixel intensity at a specified distance and direction, making it useful for deriving textural features (e.g., contrast, correlation, energy, and homogeneity).	GLCM is a matrix where each element represents the frequency of pixel intensity pairs occurring at a specified spatial relationship (distance and angle). It captures the joint probability of pixel intensity levels to analyze texture.
Gray level run length matrix (GLRLM)	Quantifies the distribution of consecutive pixels (runs) having the same gray-level intensity in a particular direction (e.g., horizontal, vertical, diagonal).	GLRLM is a matrix where each entry represents the number of runs of a specific length at a particular gray level. A run is defined as a sequence of consecutive pixels with the same intensity in a given direction.
Gray level size zone matrix (GLSZM)	Quantifies texture by measuring the size and uniformity of connected regions (zones) with the same gray-level intensity in the image, making it useful for assessing homogeneity.	GLSZM is a matrix in which each element represents the number of connected regions (zones) of a specific size and gray level, where zones are clusters of neighboring pixels sharing the same intensity.

Supplementary Table S3. Brief definitions and descriptions of selected radiomic features in conjunctiva photos for anemia

Delineated area	Radiomic feature	Description	Mathematical definition
**Palpebral conjunctiva**	Wavelet-H, GLDM, gray level nonuniformity (#1)	Captures the variability of gray levels in high-frequency regions, reflecting texture complexity.	Sum of squared gray levels divided by the total number of gray level occurrences.
	LoG, GLDM, dependence nonuniformity (#2)	Measures variability in dependent pixel groups with higher values indicating more diverse textures.	Sum of squared counts of dependency within low-pass filtered regions.
	Wavelet-L, GLDM, dependence nonuniformity (#3)	Emphasizes larger, smoother regions through nonuniformity measurement in low-frequency components.	GLDM dependence nonuniformity, derived from low-frequency wavelet components.
	Wavelet-H, GLCM, informational measure of correction 2 (#4)	Quantifies the complexity of the texture.	Correlation between the probability distributions
	Wavelet-H, GLRLM, short run emphasis (#5)	Focuses on short consecutive sequences (runs) of similar intensity values, highlighting fine texture details.	Sum of inverse run lengths, assigning greater weight to shorter runs.
	Wavelet-H, GLDM, dependence nonuniformity (#6)	Captures high-frequency details using wavelet high-pass filtering, similar to dependence nonuniformity.	Sum of squared counts of dependent pixels in high-pass components.
	Wavelet-H, GLDM, dependence nonuniformity normalized (#7)	Emphasizes high-frequency components using normalized measure of dependence nonuniformity.	Dependence nonuniformity normalized by the total number of dependent pixels.
	LoG, GLSZM, zone variance (#8)	Measures the variance in zone sizes, reflecting the distribution of homogeneous regions.	Variance of zone sizes within the matrix.
	LoG, GLDM, dependence nonuniformity (#9)	Measures the variability in the size of dependent groups of pixels with higher values indicating greater heterogeneity.	Sum of squared counts of dependent pixels divided by the total number of dependent pixels. Dependent pixels are defined as the number of connected pixel within a preset distance.
	LoG, GLSZM, large area high gray level emphasis (#10)	Emphasizes large, high-intensity regions, indicating a broad homogeneous focal area.	Sum of squares of high gray-level zone sizes divided by the total number of zones.
**Bulbar conjunctiva**	Wavelet-H, GLDM, dependence nonuniformity normalized (#1)	Captures small structural patterns in high-pass regions through normalized dependence nonuniformity.	GLDM dependence nonuniformity, normalized for high-frequency components.
	Wavelet-H, GLSZM, zone percentage (#2)	Measures the proportion of small zones within high-pass filtered image areas.	Count of zones below a specified threshold divided by the total number of zones.
	Wavelet-L, first order, interquartile range (#3)	Measures the spread of the middle 50% of intensity values, emphasizing larger structures in low-pass filtered regions.	Difference between the 75th and 25th percentile intensity values.
	LoG, GLSZM, zone variance (#4)	Measures variance in zone size, indicating the spread of uniform areas.	Variance of zone sizes in GLSZM.
	LoG, GLSZM, size zone nonuniformity normalized (#5)	Indicates structural uniformity through a normalized measure of zone size variability.	Sum of squared zone sizes divided by the total number of zones.
	LoG, first order, maximum (#6)	Captures the highest intensity value in the image after applying LoG filtering.	Maximum pixel intensity in the region of interest.
	LoG, GLDM, dependence nonuniformity normalized (#7)	Detects fine structural variations through normalized dependence nonuniformity.	GLDM dependence nonuniformity, normalized by total dependency.
	Wavelet-H, GLSZM, large area emphasis (#8)	Emphasizes large areas of similar intensity within high-pass filtered image regions.	Sum of squares of large zone sizes within high-frequency wavelet bands.
	LoG, GLCM, correlation (#9)	Measures the linear dependence between gray levels of neighboring pixels.	Calculated from the mean and variance of intensity values in GLCM.
	LoG, GLRLM, run length nonuniformity normalized (#10)	Indicates variability in run lengths through a normalized measure of run length nonuniformity.	Sum of squared run lengths divided by the total number of run lengths.

Supplementary Table S4. Multivariate linear regression coefficients and partial correction correlation coefficients for associations between blood Hgb levels and selected radiomic features

	Blood Hgb level [g dL^{-1}]	
**Palpebral conjunctiva**	**Slope coefficient (p-value)**	**Partial correlation coefficient (p-value)**
Wavelet-H, GLDM, gray level nonuniformity (#1)	0.18 (0.000)	0.11 (0.000)
LoG, GLDM, dependence nonuniformity (#2)	0.18 (0.000)	0.11 (0.000)
Wavelet-L, GLDM, dependence nonuniformity (#3)	0.17 (0.000)	0.10 (0.000)
Wavelet-H, GLCM, informational measure of correction 2 (#4)	0.06 (0.000)	0.04 (0.000)
Wavelet-H, GLRLM, short run emphasis (#5)	–0.09 (0.000)	–0.05 (0.000)
Wavelet-H, GLDM, dependence nonuniformity (#6)	0.18 (0.000)	0.11 (0.000)
Wavelet-H, GLDM, dependence nonuniformity normalized (#7)	0.03 (0.058)	0.02 (0.058)
LoG, GLSZM, zone variance (#8)	0.06 (0.000)	0.04 (0.000)
LoG, GLDM, dependence nonuniformity (#9)	0.18 (0.000)	0.11 (0.000)
LoG, GLSZM, large area high gray level emphasis (#10)	0.06 (0.000)	0.04 (0.000)
**Bulbar conjunctiva**	**Slope coefficient (p-value)**	**Partial correlation coefficient (p-value)**
Wavelet-H, GLDM, dependence nonuniformity normalized (#1)	0.09 (0.000)	0.05 (0.000)
Wavelet-H, GLSZM, zone percentage (#2)	–0.10 (0.000)	–0.06 (0.000)
Wavelet-L, first order, interquartile range (#3)	–0.09 (0.000)	–0.06 (0.000)
LoG, GLSZM_zone variance (#4)	0.10 (0.000)	0.06 (0.000)
LoG, GLSZM, size zone nonuniformity normalized (#5)	–0.06 (0.000)	–0.04 (0.000)
LoG, first order_maximum (#6)	–0.15 (0.000)	–0.09 (0.000)
LoG, GLDM, dependence nonuniformity normalized (#7)	0.10 (0.000)	0.06 (0.000)
Wavelet-H, GLSZM, large area emphasis (#8)	0.08 (0.000)	0.05 (0.000)
LoG, GLCM, correlation (#9)	0.11 (0.000)	0.07 (0.000)
LoG, GLRLM_run length nonuniformity normalized (#10)	–0.06 (0.000)	–0.04 (0.000)

Supplementary Data S1
The radiomic and clinical dataset related to the palpebral conjunctiva.

Supplementary Data S2
The radiomic and clinical dataset related to the bulbar conjunctiva.
